# Unusual Gross Pneumocephalus and Pneumoperitoneum after VP Shunt Surgery: A Case Report

**Published:** 2015-04-01

**Authors:** Khanolkar A, Sarin YK

**Affiliations:** Department of Pediatric Surgery, Maulana Azad Medical College, New Delhi-110002

**Keywords:** Pneumocephalusa, Pneumoperitoneum, VP shunt, Complications

## Abstract

We report an unusual case where a two-month infant developed a simultaneous and spontaneous pneumocephalus and gross pneumoperitoneum along with progressive surgical emphysema after VP shunt procedure.

## CASE REPORT

A male neonate born at term at home through normal vaginal delivery was brought with ruptured lumbosacral meningomyelocele soon after birth; he had no neurological deficit. Laminectomy and repair of meningo-myelocoele was done on day 1 of life. The postoperative recovery was satisfactory. He was discharged on oral acetazolamide 80mg thrice daily. During follow up, within a span of 3 weeks, his head circumference increased from 34cm to 39 cm. He also had an umbilical hernia with a 1cm fascial defect. 

On investigating during present admission, patients haemogram and kidney function test were within normal limits. 2D ECHO- showed OS-ASD with left to right shunt. Cranial ultrasonography showed dilated lateral ventricle with V/H ratio right 0.531(2.5:4.7) and left 0.55 (2.5:4.5); 3rd and 4th ventricles normal, bilateral caudothalamic groove normal and posterior fossa structures grossly normal.

VP shunting was done on the right side; umbilical hernia was also repaired concomitantly. The immediate postoperative recovery was satisfactory. Cranial ultrasonography done on postoperative day two showed bilateral ventricles dilated with V/H ratio increased on both left (2.3:5.0) and right (2.2:5.0); VP shunt tip within the right lateral ventricle.

From fourth postoperative day, the baby started having progressively increasing abdominal distension. He was otherwise afebrile, vitally stable and feeding well. He was noted to have wound infection of the cranial incision. He developed surgical emphysema along the subcutaneous tunnel of VP shunt and the scalp, which was released by opening couple of the sutures along the incision line on scalp. The abdominal distension was progressively increasing. X-ray abdomen showed gross pneumoperitoneum (Fig. 1). CT head showed bilateral ventricles dilated with VP shunt in situ with small extra-axial haemorrhage with air fluid levels (Fig. 2). Antibiotics were upgraded. All the while, the neonate did not show any signs of meningitis or peritonitis. 

**Figure:**
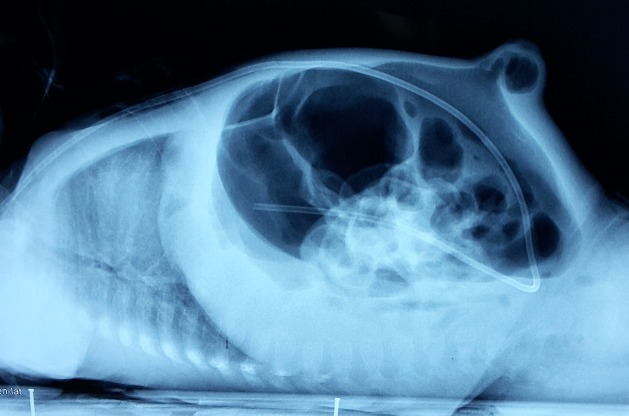
Figure 1: Abdominal roentgenograms showing gross pneumoperitoneum.

**Figure:**
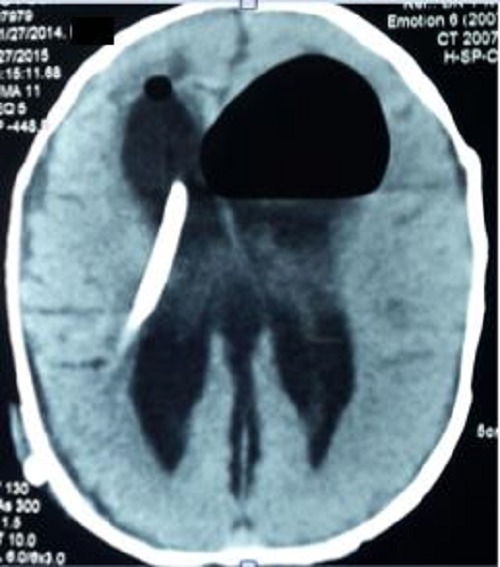
Figure 2: CT head showed air-fluid levels in both ventricles.

He was reoperated on 8th postoperative day and the infected VP Shunt was removed. Abdomen was also explored through the umbilical incision. Lot of air gushed out; however there was no evidence of any contamination; the bowel was healthy.

Postoperatively, the neonate showed improvement, abdominal distension settled. He was discharged after 5 days of observation on breast feeding, oral antibiotics, and oral acetazolamide. He was advised weekly check up with measurement of head circumference. We have planned for reinsertion of VP shunt on left side in case of progressive hydrocephalus.

The VP Shunt tip culture showed no growth but CSF culture sent showed growth of E-Coli.

## DISCUSSION

There have been several reports of complications of VP Shunt procedure. Most commonly reported are the abdominal complications with incidence in the range of 5-25% [1], which include perforation of bowel [2], pseudocyst formation [3] , distal migration of the shunt into abdominal organs and extrusion of shunt [4-6]. Intracranial pneumocephalus is mostly seen when air enters through the cranial base because of iatrogenic connection, congenital fistula, trauma or thinning of cranial base [7], while benign pneumocephalus and pneumoperitoneum is a known condition after any abdominal surgery [8]. Jea et al in 2005 [8] suggested that the air introduced under pressure during the percutaneous gastrostomy procedure through endoscope, went retrograde through the VP shunt into the ventricles causing pneumocephalus. 

In our case, we have ruled out the possibility of any bowel perforation, as there was no contamination of the peritoneum. Lung perforation was also ruled out as there was no pneumothorax. We believe there could be two possibilities; a facultative anaerobic gas forming organism infection of the shunt i.e. E-coli seen present in the CSF culture which explicates the presence of gas in the ventricles as well as the peritoneum, nevertheless, it does not explain massive volume of air; other possible reason could be the surgical site wound infection on the scalp wound requiring opening of couple of sutures that might have caused surgical emphysema and air entering into the ventricles through the burr hole and then into the peritoneum through the shunt.

To conclude, we reported an unusual case of spontaneous pneumocephalus and pneumoperitoneum after a VP shunt surgery, the etiology of which is obscured and open to debate.

## Footnotes

**Source of Support:** Nil

**Conflict of Interest:** The author is editor of the journal but he is not involved in decision making of the manuscript.

